# Proteomics in asthma: the clinicians were right after all, were not they?

**DOI:** 10.1186/s40169-017-0170-5

**Published:** 2017-10-27

**Authors:** Anirban Sinha, Peter J. Sterk

**Affiliations:** Department of Respiratory Medicine, F5-158, Academic Medical Centre, University of Amsterdam, Meibergdreef 9, 1100 AZ Amsterdam, The Netherlands

## Abstract

Clinical disease phenotypes with underlying information of molecular and biological signatures for the same, is a prerequisite for improving medical care and developing more effective, stratified management strategies. This commentary reviews the research carried out by Cao et al. to unravel biological networks associated with different clinical categories of asthma. It finally comments on the utility of using data from multiple platforms aided by integrated systems approaches to effectively find out the obvious underlying physiological disease signatures related to clinical disease sub-types.

The key effort in today’s translational medicine is to link clinical disease entities to the rapidly increasing biological knowledge, and vice versa. This has not only opened a wealth of opportunities for pathogenetic research, but is also providing a big promise for more accurate diagnostic assessment, closer patient monitoring and better tailoring therapies. Current programs of the biggest funding institutes, such as Horizon 2020 (European Union), Personalized Medicine & New Technologies (National Institute of Health) and the China Precision Medicine Initiative, PMI (Chinese Academy of Sciences) are heavily investing into adding biological disease marker signatures to augment classical clinical characteristics and to discover and exploit the underlying cellular and molecular networks of disease. In particular, this holds for biological fingerprints as composite molecular signatures of disease in individual patients. When measured and analyzed properly [1], these signatures will translate systems biology into systems medicine, allowing more comprehensive phenotyping of patients and thereby reshuffling disease taxonomies. The ambitions are high and are nothing less than providing a new era of healthcare and wellness by ‘data driven care’ [2].

Chronic non-communicable diseases (e.g. asthma) are an obvious target of systems medicine, because of their biological complexity and heterogeneity amongst patients [3] and the relatively ‘easy’ access to the lungs. Asthma exhibits multiple clinical presentations in child- and adulthood, of which the pathogenesis and curative therapies remain to be discovered. Several international consortia, such as the innovative medicines initiative (IMI) project U-BIOPRED (http://www.ubiopred.eu) and the severe asthma research program SARP (http://www.severeasthma.org) are currently integrating biological fingerprints with clinical features of (severe) asthma. The common hypothesis of those studies is that the biological networks underlying clinical asthma categories are diverse, thereby suggesting in-depth biological sampling to be required for adequately phenotyping asthma patients in view of personalized disease management.

The journal of translational medicine has recently published a comprehensive attempt by Cao et al. [4] to disentangle the biological networks behind clinically different categories of asthma. Notably, such studies can follow two distinct approaches: top-down (exploring the biological networks between clinically predefined patient groups) or bottom-up (unsupervised grouping of biological and/or clinical data aiding novel disease categories identification). This cross-sectional study followed both. The authors hypothesized that different presentations of asthma possess distinctive cellular and molecular signatures. Asthma was defined adequately by combining the criteria of episodic symptoms and documented variable airflow limitation. Different categories of asthma were based on distinguishable clinical presentation: classical (wheezing with/without dyspnea, cough/chest tightness), cough variant (cough as the sole presenting symptom) and chest tightness variant asthma (defined solely by chest tightness). The authors threw in sophisticated proteomics of induced-sputum supernatant, using reverse-phase HPLC/tandem mass spectrometry (MS/MS). The normalized data were analyzed using principal component analysis (PCA) and partial least squares discrimination analysis (PLS-DA).

The findings and message of the paper are rather straight forward. Namely, the authors found two proteomics-driven groups of subjects as defined by PCA and PLS-DA. Not unexpectedly, there were significant differences between patients with asthma and controls revealing differential expression of 23 amongst 1126 sputum proteins covering a large range of networks related to immunity, inflammation, protease activity, angiogenesis etc. Seven proteins in each subgroup of asthma showed significant differences with controls, separately. However, the major result of this study is represented by the absence of significant differences in sputum proteins between the three clinically defined subgroups of asthma: classical, cough and chest tightness variant asthma. This is rather unexpected, since if anything one would have anticipated, that there would have been biological differences between such divergent clinical categories of the disease.

Here we are, after laborious deep phenotyping various clinical categories of asthma, the patients merely express an “asthma” fingerprint at the sputum protein level. Despite the fact that it is widely appreciated that divergent biological networks are underlying the traditional clinical diagnosis of asthma, the harvest of the present study just confirms the common clinical labeling as “asthma”. Therefore, the clinicians may have been right after all: it is nothing more or less than asthma, is not it?

When looking more closely into the three clinical asthma groups in this study, apparently each group represented overlapping profiles of biological markers such as serum IgE, sputum, blood eosinophil counts and exhaled nitric oxide [4]. Hence, the alternative view at these data may be that the present clinical categories of classical, cough, and chest tightness variant asthma are each representing “complex” heterogeneous biological networks that are not related to “simple” clinical features such as the presence or absence of cough and chest tightness. Indeed, recent sputum data shows distinguishable molecular fingerprints (transcriptomics) amongst asthmatic patients [5]. The biologist’s view may therefore be that the clinicians are using irrelevant subgroups with regard to the biological mechanisms of asthma. It is not unlikely that clinical judgement only, lacks the tools to distinguish the molecular features of the disease. We tend to support the latter scenario, even though it does not help the patients who will rightly keep on asking the clinicians “why am I so heavily coughing, doctor?”

Part of the solution might be provided by sampling. The present study focused on sputum. There is new evidence that biological networks that are discriminative between clinical asthma categories do not match between sputum, bronchial brushings, nasal brushings and blood [6]. It could also be attributed towards inconsistency in obtaining rightly characterized clean sputum samples which is known to be a pervading concern [7]. This indicates that sampling just a single compartment doesn’t reveal the entire driving biology. This may have been the case in this study by Cao et al. and underlines the necessity of utilizing local as well as systemic samples in the deep phenotyping of asthma stitching information from different layers aided by multivariate systems approaches.

Finally, in the discussion section the authors do suggest that separate groups of proteins in sputum were differentially expressed in between the three groups of asthma, but do not specifically comment on the significance of those differences and the pathways implicated distinctively. This seems to be contradictory to the results section and the main message of the paper.

In conclusion, the present study provides new clues regarding the molecular mechanisms of asthma as compared to the healthy state. This may unravel novel drug targets that may benefit majority of patients in general but not specifically cater to the needs of stratified populations within the group. Nevertheless, it seems to be inevitable that biological sub-phenotyping within the traditional clinical categories is required on top. It may even lead to abandoning historical diagnoses such as asthma, by data driven care of ‘treatable traits’ instead [8]. This will shape precision medicine to the benefit of individual patients making it P4 (Predictive, Preventive, Participatory and Personalized).

## References

[1] McShane LM, Cavenagh MM, Lively TG, Eberhard DA, Bigbee WL, Williams PM, Mesirov JP, Polley MY, Kim KY, Tricoli JV, Taylor JMG, Shuman DJ, Simon RM, Doroshow JH, Conley BA (2013). Criteria for the use of omics-based predictors in clinical trials: explanation and elaboration. BMC Med.

[2] Sagi M, Pellet J, Roznovat J, Mazien A, Ballereau S, De Meulder B, Auffray C (2016). Systems medicine: the future of medical genomics, healthcare and wellness. Methods Mol Biol.

[3] Thamrin C, Frey U, Kaminsky DA, Reddel HK, Seely AJE, Suki B, Sterk PJ (2016). Systems biology and clinical practice in respiratory medicine: the twain shall meet. Am J Respir Crit Care Med.

[4] Cao C, Li W, Hua W, Yan F, Zhang H, Huang H, Ying Y, Li N, Lan F, Wang S, Chen X, Li J, Liu J, Lai T, Bao Z, Cao Y, Zhao Y, Huang G, Huang L, Huang Y, Wu P, Peng C, Chen Z, Chung KF, Zhong N, Shen H (2017). Proteomic analysis of sputum reveal novel biomarkers for various presentations of asthma. J Transl Med.

[5] Kuo CS, Pavlidis S, Loza M, Baribaud F, Rowe A, Pandis I, Sousa A, Corfield J, Djukanovic R, Lutter R, Sterk PJ, Auffray C, Guo Y, Adcock IM, Chung KF, U-BIOPRED Study Group (2017). T-helper cell type 2 (Th2) and non-Th2 molecular phenotypes of asthma using sputum transcriptomics in U-BIOPRED. Eur Respir J.

[6] Hekking PP, Loza MJ, Pavlidis S, de Meulder B, Lefaudeux D, Baribaud F, Auffray C, Wagener AH, Brinkman P, Lutter R, Bansal AT, Sousa AR, Bates SA, Pandis Y, Fleming LJ, Shaw DE, Fowler SJ, Guo Y, Meiser A, Sun K, Corfield J, Howarth PH, Bel EH, Adcock IM, Chung KF, Djukanovic R, Sterk PJ, U-BIOPRED Study Group (2017) Pathway discovery using transcriptomic profiles in adult-onset severe asthma. J Allergy Clin Immunol. doi:10.1016/j.jaci.2017.06.037 10.1016/j.jaci.2017.06.037 28756296

[7] McCartney JG, Jarjour NN, Castro M, Kraft M (2008). Chapter 16—assessment of airway inflammation. Clinical asthma.

[8] Agusti A, Bel E, Thomas M, Vogelmeier C, Brusselle G, Holgate S, Humbert M, Jones P, Gibson PG, Vestbo J, Beasley R, Pavord ID (2016). Treatable traits: toward precision medicine of chronic airway diseases. Eur Respir J.

